# Inhalation of Patchouli (*Pogostemon Cablin* Benth.) Essential Oil Improved Metabolic Parameters in Obesity-Induced Sprague Dawley Rats

**DOI:** 10.3390/nu12072077

**Published:** 2020-07-13

**Authors:** Seong Jun Hong, Jinju Cho, Chang Guk Boo, Moon Yeon Youn, Jeong Hoon Pan, Jae Kyeom Kim, Eui-Cheol Shin

**Affiliations:** 1 Department of Food Science, Gyeongnam National University of Science and Technology, Jinju 52725, Korea; 01028287383a@gmail.com (S.J.H.); aacho7@hanmail.net (J.C.); dbs7987@naver.com (C.G.B.); ringspot@naver.com (M.Y.Y.); 2 Department of Behavioral Health and Nutrition, University of Delaware, Newark, DE 19716, USA; jhpan@udel.edu (J.H.P.); jkkim@udel.edu (J.K.K.)

**Keywords:** *Pogostemon cablin* Benth., essential oil, volatile compounds, blood pressure, anti-obesity

## Abstract

This study investigated effects of patchouli essential oil (PEO) inhalation on metabolic parameters. First, to characterize aromatic compounds in PEO, solid-phase microextraction-gas chromatography/mass spectrometric detection was employed in which 19 aromatic compounds were identified. In GC-olfactometry analysis, linalool, *α*-patchoulene, and *β*-patchoulene were found to be the constituents exhibiting the highest similarity to the aromatic compounds in patchouli. In an animal experiment using Sprague Darley rats, groups with PEO inhalation had a reduced food intake compared to the control group. Additionally, body weight was lower in the obesity-induced animal model exposed to PEO inhalation than the group without PEO. However, we found no significant difference in organ weights between groups. In our serum analysis, high-density lipoprotein cholesterol was significantly higher in the PEO inhalation groups, while low-density lipoprotein cholesterol content was highest in the positive control group, suggesting that inhalation of the aromatic compounds present in patchouli may improve cholesterol profile. In addition, leptin levels were reduced in the groups treated with PEO inhalation, which explains the differences in food intake and body weight gains. Last, animal groups exposed to PEO inhalation showed a relatively lower systolic blood pressure which suggests that inhalation of PEO (or aromatic compounds therein) may assist in regulating blood pressure. Collectively, our data demonstrate that the inhalation of PEO influenced certain markers related to metabolic diseases, hence provide basic data for future research as to preventive/therapeutic applications of PEO as well as their aromatic constituents.

## 1. Introduction

According to the World Health Organization, the incidence of obesity has increased approximately 3-fold during the 2010s compared to the 1970s. Given complications, it is not surprising that obesity is classified as a non-infectious disease [1]. Obesity may induce cardiovascular disease via increased serum levels of low-density lipoprotein cholesterol (LDL cholesterol), total cholesterol (TC), serum leptin, and triglyceride (TG). Obesity may also induce other metabolic implications including hypertension, reduced serum high-density lipoprotein cholesterol (HDL cholesterol), and increased waist circumference [2]. Obesity primarily results from excessive calorie consumption (increased food intake) and/or insufficient calorie expenditure (reduced metabolic and physical activity). Secondary causes may be attributed to a genetic or endocrine dysfunction, such as hypothyroidism, insulin resistance, and hyperleptinemia [1].

Olfactory recognition is a set of processes comprising the detection, determination, and discrimination of odor. The process begins when volatile aromatic compounds bind to receptors on olfactory neurons that stimulate peripheral nerves. Subsequently, the stimuli are transported to the brain one after another [3]. The binding of volatile aromatic compounds to the olfactory receptors is specific, such that each receptor is compatible with a particular aromatic compound, and here, a complex union is formed among the receptors that allow humans to determine and discriminate odors [4,5]. Through the aforementioned series of processes, humans can distinguish thousands of odors, while recognizing each simultaneously [6]. It is demonstrated that recognized odor is able to stimulate the central nervous system (CNS) to engage in energy metabolism by regulating food intake, autonomic nerves, and lipolysis [7,8,9]. For instance, Yamamoto et al. [10] showed reduced food intake and weight gain in mice following the inhalation of *Osmanthus fragrans* odor; the odor increased pro-opiomelanocortin/cocaine- and amphetamine-regulated transcript (POMC/CART) expression and α-melanocyte-stimulating hormone (α-MSH) secretion.

CNS controls the autonomic nervous system (ANS) to influence sympathetic and parasympathetic nerve activation as well as the secretion of hormones such as leptin and circulating insulin related to the suppression of food intake. CNS thus regulates food intake [7]. Peripheral adipocytes produce leptin, where an increase in leptin level leads to the feeling of satiety. As leptin passes through the blood–brain barrier, there is an associated increase in the expression of POMC and CART neurons in the arcuate nucleus of the hypothalamus and reduced expression of prepro-orexin and agouti-related protein (AgRP) neurons. This results in a decrease in neuropeptide Y (NPY) secretion but an increase in α-MSH secretion, thereby increasing satiety [9]. However, in obesity, food intake is not reduced despite a rise in leptin level, a phenomenon referred to as leptin resistance [11,12].

Patchouli (*Pogostemon cablin* Benth.) is a plant belonging to the Lamiaceae family. It is frequently used for essential oils products [13,14]. The plant is mainly cultivated in Southeast Asia and is commercially used in perfumes and aromatherapy [14,15]. Patchouli essential oil (PEO) recently received much attention due to its diverse bioactivity including anti-inflammatory effects, antiviral activities, antioxidant effects, wound healing, and inhibitory effects on lipid accumulation [15,16,17]. Patchouli has also been reported to suppress food intake and weight gain [18], while the major volatile compounds of PEO were characterized such as patchouli alcohol, *α*-patchoulene, and *β*-patchoulene [16]. Despite previous studies, however, effects of the inhalation of PEO on metabolic disease related markers have not sufficiently been examined. Therefore, this study analyzed the aromatic compounds of PEO, while monitoring the changes in hormone, cholesterol profiles, and other metabolic parameters (e.g., body weight, height, food intake, organ weight, blood pressure) in obesity-induced rats exposed to PEO.

## 2. Materials and Methods

### 2.1. Essential Oil

The PEO was purchased from the Aroma Care Solution (Helga Stolz GmbH Co., Grafenwoerth, Austria). Upon received, the product was stored at 4 °C before the experiments to minimize any changes in the chemical compositions and bioactivities of the essential oil.

### 2.2. Volatile Compounds and Odor Description

Volatile aromatic compounds in PEO were profiled through headspace analysis using the solid phase micro-extraction (SPME) fiber (Supelco Co., Bellefonte, PA, USA) coated with 100 µm poly-dimethylsiloxane. One gram sample was placed in a sampling bottle, which was subsequently sealed with an aluminum cap. The sampling was based on exposure to the headspace of the sample preheated at 60 °C. The SPME fiber to which the aromatic compounds adhered to after sampling were analyzed using gas chromatography-mass-spectrometry (GC/MS: Agilent 7890A & 5975C, Agilent Technologies, Santa Clara, CA, USA) that was interfaced with an HP-5MS column (30 m × 0.25 mm i.d. × 0.25 µm film thickness). The analysis conditions were as follows: retention for 5 min at oven temperature 40 °C; temperature increase to 200 °C at 5 °C/min; injector temperature 220 °C; flow rate of helium as the carrier gas at 1.0 mL/min; split ratio 1:10. Each of the separated compounds on the total ionization chromatogram was identified in reference to the mass spectrum library. The aromatic compounds in each sample were expressed in the relative ratio (%) of their respective peak area by converting the peak area to percentage based on the total peak area as 100%.The olfactory detector port (ODP) was used to examine the characteristics of each separated aromatic compound sensed by the human olfactory organ. Concertedly, the retention time and intensity of odor were measured and recorded using a sensor that divides the intensity into four levels [19].

### 2.3. Animal Care and Experimental Design

This study was conducted in compliance with the Animal Protection Act and following the approval of the Institutional Animal Care and Use Committee (Document #: IACUC-4) of the Gyeongnam National University of Science and Technology. The laboratory animals used in this study were 4-week old male Sprague Dawley rats. A total of 36 rats were purchased from the Coretec Co. (Busan, Korea). After a week acclimation, animals were randomly assigned to groups, and maintained for 12 weeks. To be specific, the rats were randomly divided into groups: the normal diet fed control group (N; *n* = 6) was given a normal diet and 30-min inhalation of distilled water (DW) for 12 weeks; the high-fat diet (HFD) fed control group (H; *n* = 6) was given a 45% HFD and 30-min inhalation of DW for 12 weeks; the rest of the groups were all given an HFD to induce obesity-phenotypes. The HFD fed group with low dose PEO during the induced period (H-LPI, *n* = 6) was given 30-min inhalation of 0.3% PEO for 12 weeks. The HFD fed group with high dose PEO during the induced period (H-HPI, *n* = 6) was given 30-min inhalation of 1% PEO for 12 weeks; the HFD fed group with low dose PEO during the treatment period (H-LPT, *n* = 6) was given 30-min inhalation of 0.3% PEO only for the first 6 weeks; the HFD fed group with high dose PEO during the treatment period (H-HPT, *n* = 6) was given 30-min inhalation of 1% PEO only for the last 6 weeks, thus constituting 6 groups in total. Body weight and food intake were measured at a predetermined time each week.

After the 16 h fasting period before dissection, rats were anesthetized by ether. A syringe dipped into 20 mg of ethylenediaminetetraacetic acid was used to extract approximately 3 mL of blood from the heart. Subsequently, rats were sacrificed as the aorta was near the cut to the spinal cord. The collected blood was left to stand in ice for 30 min; then, serum was isolated by centrifugation at 3000 rpm for 30 min. Next, brain, heart, kidney, liver, white adipose tissue (WAT), and brown adipose tissue (BAT) were extracted, and serum and organs were stored in a −80 °C freezer.

### 2.4. Serum Biomarker Analyses

Serum concentration levels of TC, HDL cholesterol, and TG were measured using commercial kits (Asan-T-CHO-Lq Reagents, Asan Pharm Co., Seoul, Korea). The aspartate transaminase (AST) and alanine transaminase (ALT) were measured using the AST kit and the ALT kit (Asan-SET GOT-Lq Reagents, Asan Pharm Co., Seoul, Korea) according to the manufacturer’s instruction. The serum leptin was measured using the ELISA kit (Bertin Technologies Co., Montigny le Bretonneux, France).

### 2.5. Blood Pressure Assessment

Changes in blood pressure and pulse of rats were measured using an animal blood pressure meter (BP-2000, Visitech systems Co., Apex, NC, USA). The measurements were taken from tails of the rats at the predetermined day of week and time at weeks 1, 6, and 12. For each rat, 30 measurements were taken, including 10 preliminary and 20 main tests, regarding the systolic blood pressure (SBP), diastolic blood pressure (DBP), and pulse. The mean of 6 measurements that showed the least deviation and excluding the highest and lowest values was recorded and used for statistical analyses.

### 2.6. Statistical Analyses

The experiments in this study were repeated three times, from which the mean and standard deviations (SD) were estimated and used to present the result values. Our paired groups were non-parametrically compared using the Friedman test with the chi-square distribution followed by Dunn’s post test. *p*-value less than 0.05 was considered statistically different (SAS Institute Inc., Cary, NC, USA).

## 3. Results and Discussion

### 3.1. Volatile Compounds

Aromatic compounds in PEO were analyzed using the GC/MS method, and the results of identified aromatic compounds based on the ODP are presented in the Table 1. The volatile compounds in PEO included 6 alcohols, 10 hydrocarbons, 2 heterocyclics, and 1 ketone. The patchouli odor was found in linalool, *α*-patchoulene, and *β*-patchoulene, while β-caryophyllene and citronellol showed a forest-like odor. Although the odor could not be detected based on the ODP, patchouli alcohol is known as an essential aromatic compound in PEO [16,20]. *β*-selinene and carvone did not show the patchouli odor, but they were found to be volatile compounds with a relatively high percentage in PEO. Above all, patchouli alcohol, *α*-patchoulene, and *β*-patchoulene are the volatile compounds accounting for the highest percentage in PEO. The structure of these compounds has been known for several decades, and previous studies have reported on the detection of *α*-patchoulene using GC/MS [16]. It is known that patchouli alcohol exhibits antimicrobial activity and anti-inflammatory effect, as well as immunomodulation and improvement in rat paw edema [21]. Similarly, *β*-patchoulene is known to prevent inflammatory reaction and neovascularization, thereby improving stomach ulcers [22], while linalool has soothing effects with known influence on increasing sleep duration and decreasing body temperature upon inhalation [23]. Carvone is found in the essential oil of *Carum carvi* L. (caraway) and is known for its anticonvulsant, anti-inflammatory, and antimicrobial properties [24]. Citronellol is one of the primary compounds found in citronella oil, where inhalation is effective in lowering BAT temperature [25]. Moreover, *E*-caryophyllene is one of the major compounds in several essential oils, having positive effects by reducing inflammation, acute pain, and chronic pain as well as regulating insulin secretion [26,27,28]. *β*-selinene is found in various essential oils, including PEO; an oil enriched with *β*-selinene shows anti-inflammatory and pain relief effects as well as lowering body temperature, in addition to being a powerful antioxidant [29]. Tsuji et al. [30] reported that inhalation of limonene, a primary compound in grapefruit oil, elicits hypoglycemic effects. All above evidence in literature indicate that inhalation of volatile compounds may influence physiological functions in vivo.

### 3.2. Food Intake, Body Weight, and Length

No behavioral abnormality or noticeable symptomatology was found concerning PEO inhalation. Changes in food intake during the study period is shown in the Table 2. The food intake was the highest in group N animals. The H-LPI and H-LPT groups did not show any significant differences among HFD fed groups, whilst the H-LPI and H-LPT showed a decreased food intake compared to the group H. The high dose groups (H-HPI and H-HPT) showed a trend of decrease in food intake, although statistical significance was not achieved.

**Figure 1:**
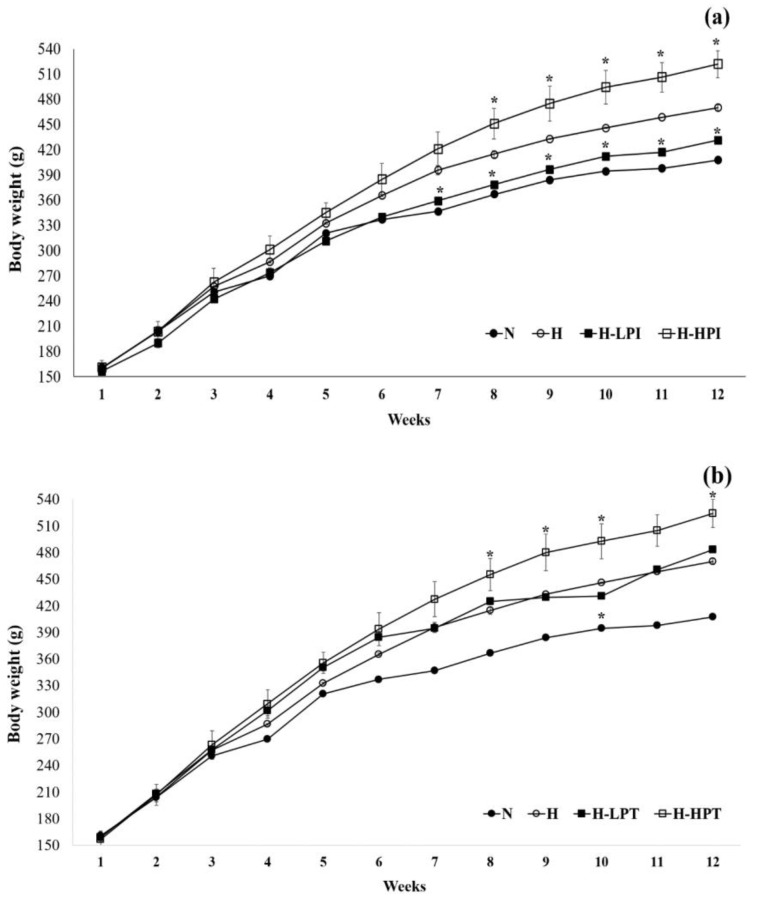
Changes in body weight between groups. (**a**) Animal groups fed high fat diet with inhalation of patchouli essential oil (PEO) during the induced period (7~12th weeks), and (**b**) animal groups fed high fat diet with inhalation of PEO during the total periods (1~12th weeks). Symbol (*) corresponds, at each time point, the significant difference among groups H versus inhalation of PEO by the non-parametric Friedman test followed by Dunn’s test (*p* < 0.05).

**Figure 2:**
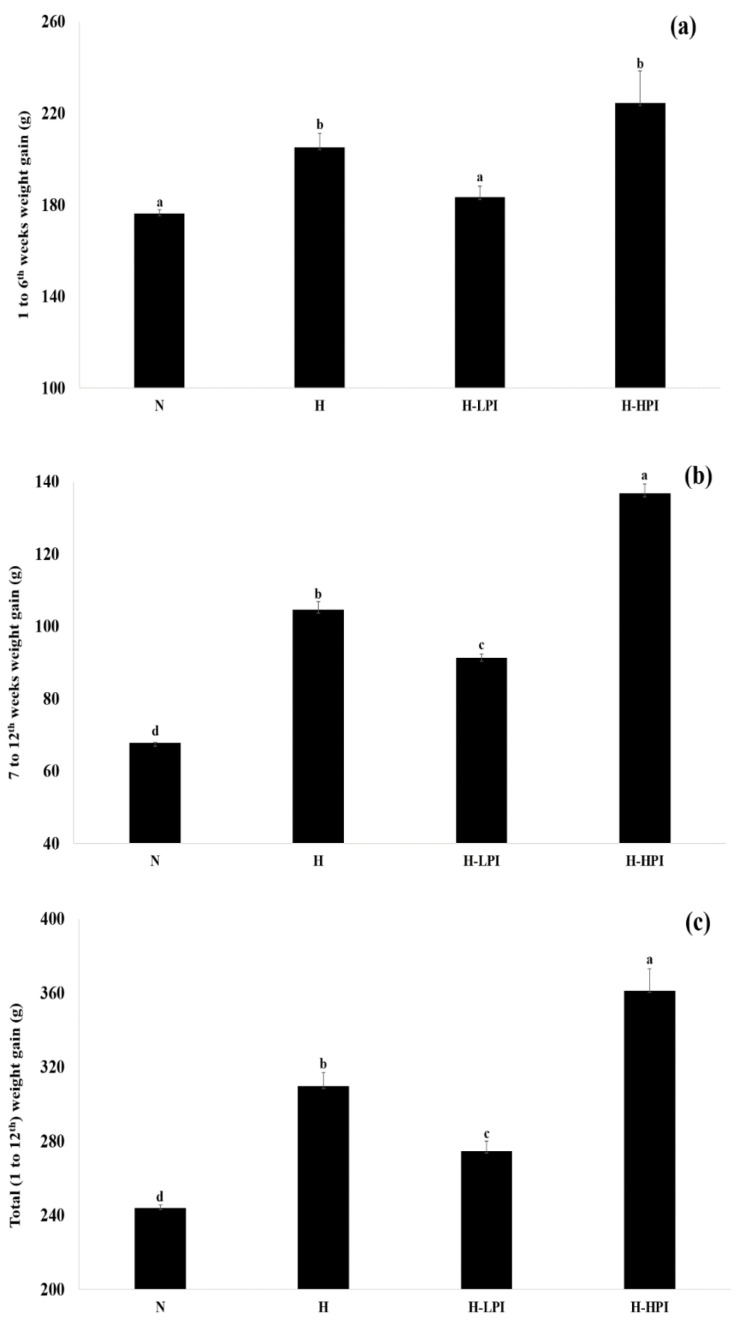
Comparisons of body weight gains between groups (1~12th weeks). (**a**) Weight gain during the period from 1 to 6th weeks, (**b**) weight gain during the period from 7 to 12th weeks, and (**c**) total weight gain during the periods from 1 to 12th weeks. Mean with different letters (a–d) corresponds the significant difference determined through the non-parametric Friedman test followed by Dunn’s test (*p* < 0.05).

**Figure 3:**
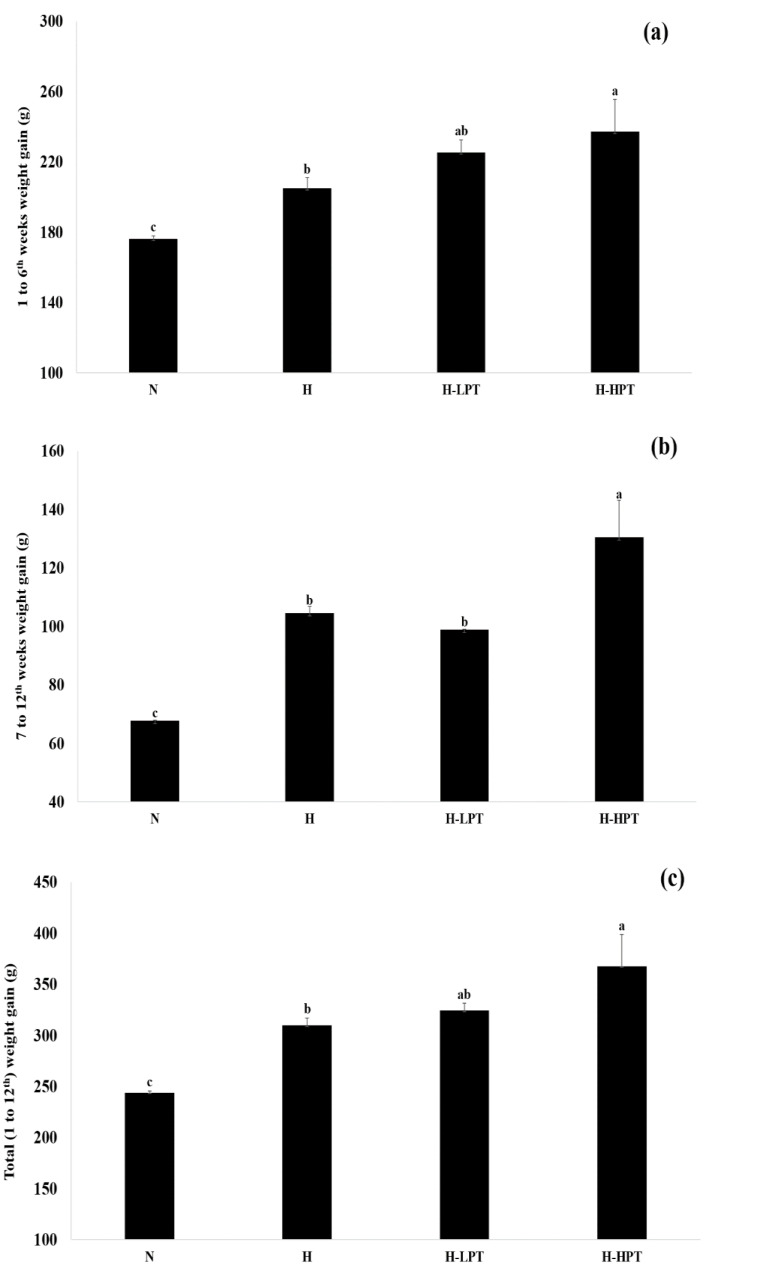
Comparisons of body weight gains between groups during the entire intervention period (from 1 to 12th weeks). (**a**) Weight gain during the period from 1 to 6th weeks, (**b**) weight gain during the period from 7 to 12th weeks, and (**c**) total weight gain during the periods from 1 to 12th weeks. Mean with different letters (a–c) corresponds the significant difference calculated by the non-parametric Friedman test followed by Dunn’s test (*p* < 0.05).

Changes in body weight during the intervention period is summarized in Figure 1, Figure 2 and Figure 3. The group N showed the lowest body weight amongst all groups while, among the HFD fed groups, the H-LPI had the lowest body weight (*p* < 0.05). The H-HPI and H-HPT group animals showed ≥10% increase in body weight compared to the H group (*p* < 0.05). Lastly, the H-LPT group rats showed a trend of increase in body weight compared to the group H during the first 6 weeks without PEO inhalation; however, during the last 6 weeks with PEO inhalation, the body weight showed a lowered rate of increase compared to the group H.

According to the U.S. Food and Drug Administration criteria, an average ≥5% increase in body weight indicates obesity. In addition, the analysis of body components using the dual-energy X-ray absorptiometry in a preliminary study demonstrated that body fat was reduced without notable muscle loss in the H-LPI group. In contrast, the group with inhalation of high dose PEO showed an approximate ≥10% increase in body fat compared to group H, accompanying muscle loss (Data not shown). Concerning lipid accumulation, studies have reported a positive correlation with insulin levels in the blood [31], suggesting that the group with inhalation of high dose PEO developed accelerated obesity. Furthermore, the reduced body weight in group H-LPI was thought to be due to the suppressed accumulation of body fat. The suppressed body weight in group H-LPT after inhalation of PEO was also thought to be due to the suppressed accumulation of body fat. Heo et al. [18] showed that, despite a decreasing mean value of serum leptin levels in rats following PEO inhalation, food intake decreased, which implied a decrease in leptin resistance. According to previous studies, serum leptin levels and body weight show a positive correlation. However, when obesity occurs, the consequent leptin resistance is thought to diminish the correlation between serum leptin levels and food intake. The decreasing trend of food intake in group H-LPI may be due to a decrease in leptin resistance, while the same trend in the other groups with PEO inhalation is presumed to be due to the reduced leptin levels and a decrease in leptin resistance [12]. Furthermore, in Table 1, citronellol is one of the major compounds in PEO and a volatile compound known to inhibit obesity by reducing food intake [25], while patchouli alcohol, *α*-patchoulene, and *β*-patchoulene, which account for the largest proportion of PEO and release the PEO odor, are thought to influence the decrease in food intake by stimulating the hypothalamus and regulating serum leptin levels.

**Figure 4:**
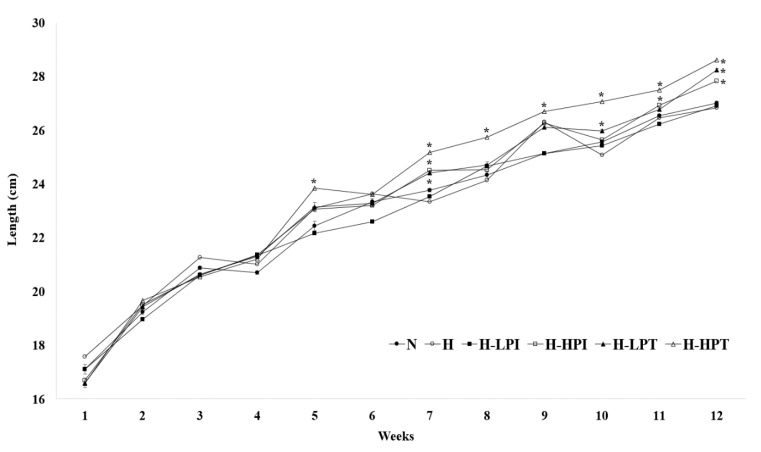
Changes in body length between groups. Symbol (*) corresponds the statistical difference between intervention groups versus inhalation of patchouli essential oil by the non-parametric Friedman test followed by Dunn’s test (*p* < 0.05).

In the case of body length, the group H (9.2 ± 0.3 cm) showed the lowest length gain compared to the other groups with PEO inhalation (Table 2 and Figure 4). Groups H-LPI, H-LPT, and H-HPT showed a significantly lower initial length than the group H (*p* < 0.05). Body lengths, measured at the end of intervention (at 12th week), of groups H-LPI, H-LPT, and H-HPT were 27.8 ± 0.3 cm, 28.2 ± 0.5 cm, and 28.6 ± 0.3 cm, respectively, which were all statistically different than the group H (*p* < 0.05). Body length is greatly influenced by the growth hormones (GHs), as the GHs facilitate the production of insulin-like growth factor-1 (IGF-1) in the liver, thereby regulating the effects on musculoskeletal tissue or the related organs and other connective tissues that influence growth [32]. IGF-1 is similar to insulin, when it comes to amino acids sequence, and the secretion of insulin has an indirect influence on the production of IGF-1 in the liver, which also functions in cartilage formation and bone growth [33]. In addition, Yanai et al. [33] reported a positive correlation between insulin, IGF-1, and GHs. In Table 2, the PEO inhalation groups showed a significant increase in body length. This may be due to direct/indirect influences of the increased insulin secretion caused by the accumulation of lipids and volatile compounds in PEO on the production of IGF-1 [32].

### 3.3. Organ Weight

Changes in organ weight were analyzed (Table 3). Weights of the brain showed a range of 3.43 ± 0.05~4.20 ± 0.06 g/kg, while the liver weights ranged 20.25 ± 0.80~24.82 ± 0.07 g/kg. Liver weight did not show intergroup variations among the HFD fed groups except for the group H-LPI (*p* < 0.05). Compared to the other HFD fed groups, the group H-LPI showed an increase in liver weight (*p* < 0.05). Kidney weights ranged 4.58 ± 0.03~5.35 ± 0.01 g/kg. In addition, no difference in heart weight was noted among the HFD fed groups. The groups H-HPI and H-HPT had higher WAT weight compared to the other groups (*p* < 0.05). Lastly, BAT weight was lower in the group H-HPT compared to the group H, while it was not different when compared to the group H-HPI.

The metabolic characteristics of adipose tissues are different depending upon the specific area of distribution; and WAT is one of the indicators of visceral fat [34]; increase in visceral fat is positively correlated with abdominal fat content. In addition, the accumulation of excess visceral adipocytes promotes release of free fatty acids and facilitates the synthesis of very-low-density lipoprotein cholesterol and gluconeogenesis in the liver. This causes an abnormal insulin signaling and glucose metabolism whereby insulin resistance arises [31]. It was also reported that a significant increase in WAT leads to obesity, cardiovascular disease, metabolic syndrome, and even cancers [31]. For the groups exposed to low dose PEO, the reduced body fat may explain the decreases in body weight. A loss of body fat may be due to an increase in thermogenic activation through WAT turning beige, increase in mitochondrial respiration, and/or increase in fatty acid oxidation related to the AMPK activation or inhibition of adipocytes, such as 3TL-L1 [35]. For the groups, exposed to high dose PEO, on the other hand, it is possible that the secretion of insulin, a hormone with a role in lipid accumulation [31,36], might have been increased which led to the increase in WAT (Table 3). BAT activation is involved in heat generation, which has been reported to reduce body fat [37]. Not only does an increase in BAT activity lead to heat generation, but it also results in an increased sensitivity to insulin, consequently lowering insulin resistance [37]. As shown in Table 3, BAT weight was lower in the group H-HPT compared to the group H, while it was not different when compared to the group H-HPI.

### 3.4. Serum Analysis

Serum biomarkers are presented in the Table 4. Cholesterol is divided into TC, HDL cholesterol, and LDL cholesterol. LDL cholesterol hypercholesterolemia and HDL cholesterol hypocholesterolemia have been reported as risk factors of hyperlipidemia and atherosclerosis [36]. TC was the highest in group H-LPT (166.49 ± 8.81 mg/dL), while no significant difference was observed among the other groups. HDL cholesterol contents showed an increasing trend in all groups with PEO inhalation; specifically, the contents in groups H-HPI and H-HPT showed a significant increase compared to others *(p* < 0.05). LDL cholesterol, which deposits excess cholesterol in blood vessel walls, was found to be highest in the group H (149.53 ± 8.86 mg/dL), while all other groups except H-LPT showed a significant decrease compared to the H group (*p* < 0.05). No significant change in serum TG was found among all groups. Atherogenic index and cardiac risk factor are biomarkers related to the levels of cholesterol and TG; there was a significant decrease in all groups exposed to PEO inhalation, compared to the group H (3.32 ± 0.50 mg/dL, 4.32 ± 0.50 mg/dL) (*p* < 0.05). Regarding change in leptin levels, a decreasing trend of mean value was found in all groups with PEO inhalation, compared to the group H (4204.50 ± 434.33 pg/mL). AST showed no significant intergroup difference, while ALT either displayed any significance compared to group H (85.61 ± 27.54 Karmen/mL) or showed a significant decrease as observed in the group H-LPT (*p* < 0.05).

Given that HDL cholesterol is characteristic of vascular antioxidant capacity, increasing HDL cholesterol levels enhances the body’s anti-inflammatory and antioxidant activities [38]. When LDL cholesterol increases, the risk of cardiovascular diseases, such as atherosclerosis, increases due to the accumulation of LDL cholesterol in vascular walls [31,38]. The increase in HDL cholesterol and decrease in LDL cholesterol observed in the groups with PEO inhalation, is thought to have reduced the levels of atherogenic index and cardiac risk factor, which implicates a potential fall in the risk of cardiovascular diseases [31,36,39] cholesterol.

As seen in Table 2 and Table 4, leptin levels showed a decreasing trend of mean value across all groups with PEO inhalation compared to the group H, and mean value of food intake also showed relatively a decreasing trend. Despite the decreased mean value of food intake, groups H-HPI and H-HPT showed a trend of accelerated increase in body weight following PEO inhalation and a significant increase in body weight compared to the group H (*p* < 0.05). Citronellol is one of the primary compounds in PEO. Upon PEO inhalation, it not only reduces food intake to suppress weight gain but also lowers the serum cholesterol level [25]. In line with previous studies, the PEO inhalation in this study could have led to decreased body weight and cholesterol levels. Leptin is a hormone involved in food intake regulation [9,31]. A rise in leptin levels increases the expression of POMC and CART neurons in arcuate nucleus of the hypothalamus but decreases the expression of AgRP and prepro-orexin. This influences an increased secretion in α-MSH and decreased secretion of NPY, consequently reducing food intake [9]. As such, leptin is present in high concentrations in the serum across most obesity groups; nevertheless, a lesion along the leptin signaling pathway to the hypothalamus could lead to reduced food intake, and increased energy consumption does not occur. This is referred to as leptin resistance [12,40]. Leptin resistance may lower the ability to control appetite and accelerate obesity while inducing hypertension through stimulating the sympathetic nervous [41]. Table 2 and Table 4 show that in all groups with PEO inhalation, leptin levels are relatively low compared to the group H, with a decreasing trend for mean value of food intake. Such a trend is thought to be due to a decrease in leptin resistance caused by PEO inhalation despite the relatively low level of leptin secretion [12]. AST and ALT are liver parameters, and when their levels are increased by liver toxicity, hepatocirrhosis or hepatocarcinoma may be induced. Between the groups with PEO inhalation and those without inhalation, no significant difference was found [38]. This suggests that PEO inhalation does not influence liver toxicity [38].

### 3.5. Blood Pressure

The incidence of obesity concurrently increases the incidence of metabolic syndrome such as increased blood pressure [31]. Hypertension is caused by a continuous increase in systolic blood pressure (SBP) and/or diastolic blood pressure (DBP) until they exceed normal threshold parameters. The factors that induce hypertension are leptin resistance, insulin resistance, reduced BAT activity, obesity due to visceral fat accumulation, and effects of sympathetic and parasympathetic nerves in ANS [31,37,41]. Change in blood pressure based on PEO inhalation is presented in Table 5. During the initial period, SBP did not show any significant intergroup differences, while during the obesity-induced period, mean value of SBP in the groups H-LPI and H-HPI with PEO inhalation was relatively lower than the group H. In addition, across all groups with PEO inhalation, an effect of reduced blood pressure compared to the group N was shown during the final period. Notably, group H-HPI showed significantly reduced SBP compared to group N and H (*p* < 0.05). The final SBP measurements showed an increase to 91.0 ± 3.3 and 79.7 ± 2.9 mmHg, respectively, compared to the initial SBP in group N and H. After the obesity-induced period, groups H-LPT and H-HPT with PEO inhalation showed an increase to 91.0 ± 4.9 and 85.0 ± 3.4 mmHg, respectively, indicating a similar level or a relative fall in comparison to groups N and H. Lastly, groups H-LPI and H-HPT with PEO inhalation from the initial period showed a significant increase compared to group N (*p* < 0.05) but a relative decrease compared to the group H, which implicated a suppressed increase in blood pressure caused by obesity. During the initial period, DBP did not show any significant intergroup differences, while during the obesity-induced period, DBP in groups H-LPI and H-HPT was 64.7 ± 16.8 and 65.7 ± 4.6 mmHg, indicating a significant decrease compared to the group H (110.7 ± 19.9 mmHg) (*p* < 0.05), while all groups with PEO inhalation showed a relatively lower level of DBP than the group N. During the final period, DBP was highest in group H (140 ± 36.5 mmHg), while all groups with PEO inhalation showed a lower level of DBP than group H (*p* < 0.05). Lastly, pulse rate showed a trend of gradual decrease with the growth of rats across all groups, while no significant difference was found among the HFD fed groups in the final period. Leptin is correlated with hypertension induced by obesity. Upon leptin resistance, hypertension is facilitated through increased retention time of sodium in the blood vessels or stimulated activity of sympathetic nerves [41]. The previously described reduced levels of SBP and DBP in the groups with PEO inhalation may be attributed to an improvement in leptin resistance and the reduced levels of total cholesterol and LDL cholesterol that consequently lowered the risk of cardiovascular diseases, with a hypotensive effect [31,41]. In the human clinical trial conducted by Kim et al. [19], the inhalation of *Chrysanthemum indicum* L. odor led to reduced activity of sympathetic nerves but increased activity of parasympathetic nerves, which coincided with the results of reduced SBP and DBP levels.

## 4. Conclusions

In summary, this study shows that PEO inhalation had improved metabolic parameters in the obesity-induced rats. Nevertheless, the findings are limited to the obesity-induced rat model. Accordingly, it is necessary that future studies should be conducted in order to investigate the effect(s) of inhalation of PEO in healthy animals.

## Figures and Tables

**Table 1:** Volatile compounds of patchouli essential oil by GC/MS and GC-olfactormetry (GC-O).

Major Compound	RT ^1^	RI ^2^	Mean ± SD	Odor Intensity	Odor Description	I.D. ^3^
	(min)		(µg/100 g)			
**Alcohols (6)**						
Eucalyptol	16.14	1057	44.68 ± 30.14	2	Cool	MS
Linalool	18.08	1118	12.75 ± 5.84	3	Forest, patchouli	MS/RI
Isopulegol	18.76	1142	0.49 ± 0.70	2	Bug	MS
*α*-Terpineol	20.79	1210	4.03 ± 0.64	3	Sweet	MS/RI
Citronellol	21.84	1250	4.28 ± 3.44	3	Patchouli	MS
Patchouli alcohol	32.97	>1700	2439.91 ± 2175.56	-	N.D.	MS
**Hydrocarbons (10)**						
Myrcene	14.78	1012	1.96 ± 0.71	1	Grape	MS/RI
*α*-Terpinene	15.56	1038	11.83 ± 6.26	2	Mint	MS
1,3,7-Dimethyl-3,7-octatriene	16.54	1069	0.73 ± 1.03	1	Forest	MS
Estragole	23.59	1313	0.44 ± 0.62	1	Sweet	MS
Aromadendrene	25.59	1390	1.94 ± 2.74	3	Forest	MS
*β*-Patchoulene	26.36	1421	2384.05 ± 1132.99	3	Patchouli	MS
*β*-Caryophyllene	27.14	1453	1065.62 ± 390.37	3	Patchouli	MS/RI
*α*-Patchoulene	28.62	1513	2570.51 ± 119.99	2	Cool, Flower	MS/RI
*β*-Selinene	28.89	1524	414.90 ± 350.10	2	Forest	MS
*β*-Humulene	30.04	1573	9.51 ± 13.45	2	Forest	MS
**Heterocyclics (2)**						
2,5-Diethyltetrahydro-5-diethyltetrahydrofuran	11.41	911	2.96 ± 3.63	2	Cool	MS
Limonene oxide	18.68	1139	0.12 ± 0.17	3	Sweet	MS/RI
**Ketone (1)**						
Carvone	22.55	1275	479.75 ± 493.67	4	Mint	MS

Data are given as mean ± SD values from experiments performed in duplicate. ^1^ RT: retention time, ^2^ RI: retention index, ^3^ I.D.: identification.

**Table 2:** Effects of patchouli essential oil inhalation on food intake and body length.

Group	Food Intake (g/day)	Initial Length (cm)	Final Length (cm)	Length Gain (cm)
N	18.18 ± 0.02 ^a^	17.1 ± 0.1 ^a,b^	27.0 ± 0.1 ^b,c^	9.9 ± 0.1 ^c^
H	16.90 ± 1.69 ^a,b^	17.6 ± 0.1 ^a^	26.8 ± 0.4 ^c^	9.2 ± 0.3 ^d^
H-LPI	14.88 ± 0.23 ^b^	17.1 ± 0.3 ^a,b^	26.9 ± 0.2 ^c^	9.8 ± 0.1 ^c^
H-HPI	15.97 ± 0.13 ^a,b^	16.7 ± 0.2 ^b^	27.8 ± 0.3 ^a,b^	11.1 ± 0.1 ^b^
H-LPT	15.59 ± 0.07 ^a,b^	16.6 ± 0.2 ^b^	28.2 ± 0.5 ^a^	11.6 ± 0.3 ^a,b^
H-HPT	16.74 ± 0.01 ^a,b^	16.6 ± 0.1 ^b^	28.6 ± 0.3 ^a^	12.0 ± 0.2 ^a^

Data are given as mean ± SD values from experiments performed in triplicate. Mean values with different letters within the same row are significantly different according to the non-parametric Friedman test followed by Dunn’s test (*p* < 0.05).

**Table 3:** Effects of inhalation of PEO on organ and tissue weights (g/kg).

Group	Brain	Liver	Kidney	Heart	WAT	BAT
N	4.20 ± 0.01 ^a^	24.82 ± 0.07 ^a^	5.35 ± 0.01 ^a^	2.85 ± 0.03 ^a^	37.57 ± 1.25 ^d^	0.61 ± 0.05 ^a,b^
H	3.91 ± 0.06 ^b^	20.72 ± 0.36 ^b^	5.08 ± 0.11 ^a,b^	2.55 ± 0.04 ^b,c^	58.48 ± 1.65 ^b,c^	0.72 ± 0.06 ^a^
H-LPI	4.20 ± 0.06 ^a^	23.06 ± 1.30 ^a^	5.33 ± 0.17 ^a,b^	2.71 ± 0.02 ^a,b^	57.23 ± 0.92 ^b,c^	0.60 ± 0.11 ^a,b^
H-HPI	3.43 ± 0.05 ^d^	21.07 ± 0.58 ^b^	4.58 ± 0.03 ^c^	2.45 ± 0.04 ^c^	72.10 ± 5.98 ^a^	0.59 ± 0.02 ^a,b^
H-LPT	3.50 ± 0.02 ^c,d^	20.25 ± 0.80 ^b^	4.90 ± 0.31 ^b,c^	2.52 ± 0.01 ^c^	55.46 ± 1.68 ^c^	0.66 ± 0.05 ^a^
H-HPT	3.65 ± 0.16 ^c^	20.77 ± 0.26 ^b^	4.65 ± 0.10 ^c^	2.49 ± 0.14 ^c^	64.18 ± 0.18 ^b^	0.50 ± 0.01 ^b^

Data are expressed as mean ± SD values from experiments performed in triplicate. Mean values with different letters within the same row are significantly different according to the non-parametric Friedman test followed by Dunn’s test (*p* < 0.05). WAT, white adipose tissue; BAT, brown adipose tissue.

**Table 4:** Effect of PEO on the serum profile of normal diet and HFD fed male rats.

	HDL (mg/dL)	TC (mg/dL)	LDL (mg/dL)	AI (mg/dL)	CRF (mg/dL)	LHR (mg/dL)	TG (mg/dL)	Leptin (pg/mL)	AST (Karmen/mL)	ALT (Karmen/mL)
N	28.80 ± 3.48 ^c^	148.89 ± 5.61 ^a,b^	109.50 ± 4.34 ^a^	4.20 ± 0.45 ^a^	5.20 ± 0.45 ^a^	3.83 ± 0.30 ^a^	120.26 ± 4.79 ^a^	2954.50 ± 380.31 ^a^	21.78 ± 3.29 ^a^	54.60 ± 2.47 ^b,c^
H	34.87 ± 3.79 ^c^	149.53 ± 8.86 ^a,b^	114.66 ± 9.01 ^a^	3.32 ± 0.50 ^a,b^	4.32 ± 0.50 ^a,b^	3.32 ± 0.50 ^a^	115.74 ± 5.45 ^a^	4204.50 ± 434.33 ^a^	10.16 ± 5.47 ^a^	85.61 ± 27.54 ^a,b^
H-LPI	42.54 ± 10.00 ^b,c^	136.68 ± 4.18 ^b^	73.72 ± 4.28 ^b^	2.31 ± 0.64 ^b,c^	3.31 ± 0.64 ^b,c^	1.79 ± 0.39 ^b^	137.45 ± 11.39 ^a^	4021.06 ± 1189.34 ^a^	24.73 ± 7.57 ^a^	111.68 ± 5.24 ^a^
H-HPI	59.77 ± 3.24 ^a^	144.44 ± 8.11 ^b^	90.52 ± 0.86 ^b^	1.42 ± 0.23 ^c^	2.42 ± 0.23 ^c^	1.51 ± 0.07 ^b^	118.85 ± 14.80 ^a^	3079.72 ± 850.18 ^a^	20.16 ± 5.75 ^a^	110.75 ± 1.99 ^a^
H-LPT	54.26 ± 3.65 ^a,b^	166.49 ± 8.81 ^a^	113.73 ± 6.30 ^a^	2.08 ± 0.34 ^c^	3.07 ± 0.34 ^c^	2.10 ± 0.16 ^b^	147.14 ± 58.99 ^a^	2659.33 ± 439.30 ^a^	19.69 ± 8.61 ^a^	52.21 ± 0.87 ^c^
H-HPT	57.95 ± 1.07 ^a^	145.38 ± 9.13 ^b^	87.43 ± 10.01 ^b^	1.51 ± 0.20 ^c^	2.51 ± 0.20 ^c^	1.51 ± 0.20 ^b^	144.82 ± 9.23 ^a^	3239.72 ± 252.16 ^a^	12.31 ± 1.00 ^a^	53.98 ± 4.25 ^b,c^

Data are expressed as mean ± SD values from experiments performed in triplicate. HFD: high-fat diet. Mean values with different letters within the same row are significantly different according to the non-parametric Friedman test followed by Dunn’s test (*p* < 0.05). Atherogenic index (AI) = (TC-HDL/HDL); Cardiac risk factor (CRF) = (TC/HDL); LHR = LDL/HDL × 100; TG, Triglyceride.

**Table 5:** Effect of patchouli essential oil on the systolic blood pressure, diastolic blood pressure, and pulse.

	Initial Period	Obesity-Induced Period	Final Period
Systolic (mmHg)			
N	135.7 ± 1.2 ^a,b^	218.0 ± 3.5 ^b^	226.7 ± 4.5 ^a,b^
H	156.0 ± 7.8 ^a^	248.3 ± 4.0 ^a^	235.7 ± 4.9 ^a^
H-LPI	148.7 ± 15.6 ^a,b^	197.0 ± 4.6 ^b,c^	224.0 ± 6.0 ^a,b,c^
H-HPI	148.7 ± 11.5 ^a,b^	208.0 ± 8.9 ^b,c^	211.3 ± 5.9 ^c^
H-LPT	128.7 ± 8.4 ^b^	203.7 ± 8.4 ^c^	219.7 ± 3.5 ^b,c^
H-HPT	130.7 ± 1.5 ^a,b^	193.0 ± 14.2 ^c^	215.7 ± 4.9 ^b,c^
Diastolic (mmHg)			
N	76.7 ± 5.1 ^a^	99.7 ± 6.8 ^a,b^	91.3 ± 2.1 ^b^
H	82.7 ± 5.8 ^a^	110.7 ± 19.9 ^a^	140.0 ± 36.5 ^a^
H-LPI	98.7 ± 28.2 ^a^	64.7 ± 16.8 ^b^	115.0 ± 4.4 ^a,b^
H-HPI	94.3 ± 18.1 ^a^	77.3 ± 7.0 ^a,b^	104.0 ± 7.9 ^a,b^
H-LPT	79.0 ± 4.4 ^a^	76.0 ± 15.1 ^a,b^	96.3 ± 15.9 ^a,b^
H-HPT	65.0 ± 23.3 ^a^	65.7 ± 4.6 ^b^	127.0 ± 11.5 ^a,b^
Pulse (beats/min)			
N	477.7 ± 11.4 ^a^	383.7 ± 1.5 ^a,b^	366.0 ± 1.7 ^b^
H	421.0 ± 12.5 ^b^	414.3 ± 3.8 ^a^	382.7 ± 15.5 ^a,b^
H-LPI	404.3 ± 18.1 ^b^	383.0 ± 38.1 ^a,b^	372.0 ± 18.5 ^a,b^
H-HPI	381.0 ± 19.0 ^b^	354.0 ± 22.3 ^b^	363.7 ± 24.1 ^b^
H-LPT	411.7 ± 17.1 ^b^	384.7 ± 2.1 ^a,b^	420.3 ± 18.5 ^a^
H-HPT	394.3 ± 4.0 ^b^	372.3 ± 5.1 ^a,b^	396.0 ± 24.0 ^a,b^

Data are expressed as mean ± SD values from experiments performed in triplicate. Mean values with different letters within the same row are significantly different according to the non-parametric Friedman test followed by Dunn’s test (*p* < 0.05). Obesity-induced period: 7 weeks.

## References

[1] Lee J.Y., Jeong Y., Kim C.Y. (2019). Obesity and dietary supplements for weight loss. Food Ind. Nutr..

[2] Kaplan N.M. (1989). The deadly quartet. Upper-body obesity, glucose intolerance, hypertriglyceridemia, and hypertension. Arch. Intern. Med..

[3] Firestein S. (2001). How the olfactory system makes sense of scents. Nature.

[4] Buck L., Axel R. (1991). A novel multigene family may encode odorant receptors: A molecular basis for recognition. Cell.

[5] Gaillard I., Rouquier S., Pin J.P., Mollard P., Richard S., Barnabe C., Demaille J., Giorgi D. (2002). A single olfactory receptor specifically binds a set of odorant molecules. Eur. J. Neurosci..

[6] Bushdidl C., Magnasco M.O., Vosshall L.B., Kellerl A. (2014). Humans can discriminate more than 1 trillion olfactory stimuli. Science.

[7] Riera C.E., Tsaousidou E., Halloran J., Follett P., Hahn O., Pereira M.M.A., Ruud L.E., Alber J., Tharp K., Anderson C.M. (2017). The sense of smell impacts metabolic health and obesity. Cell Metab..

[8] Shen J., Niijima A., Tanida M., Horii Y., Meada K., Nagai K. (2005). Olfactory simulation with scent of grapefruit oil affects autonomic nerves, lipolysis and appetite in rats. Neurosci. Lett..

[9] Schwartz M.W., Woods S.C., Porte D.J., Seeley R.J., Baskin D.G. (2000). Central nervous system control of food intake. Nature.

[10] Yamamoto T., Inui T., Tsuji T. (2013). The odor of Osmanthus fragrans attenuates food intake. Sci. Rep..

[11] Bengt F.B., Jens C.B. (2010). CNS leptin and insulin action in the control of energy homostasis. Ann. N. Y. Acad. Sci..

[12] Myers M.G., Cowley M.A., Münzberg H. (2008). Mechanisms of leptin action and leptin resistance. Annu. Rev. Physiol..

[13] Akhila A., Tewari R. (1984). Chemistry of patchouli oil: A review. Curr. Res. Med. Aromat. Plants.

[14] Amborse D.C.P., Annamalai S.J.K., Naik R. (2013). Effect of drying on the volatile oil yield of patchouli. Indian J. Sci. Technol..

[15] Huang Q.H., Wu X., Chen X.H., Wu J.Z., Su Z.R., Liang J.L., Li Y.C., Lai X.P., Chen J.N., Liu Y.H. (2018). Patchouli oil isolated from the leaves of *Pogostemon cablin* ameliorates ethanol-induced acute liver injury in rats via inhibition of oxidative stress and lipid accumulation. RSC Adv..

[16] Beek V.T.A., Joulain D. (2017). The essential oil of patchouli, *Pogostemon cablin*: A review. Flavour Fragr. J..

[17] Yang X., Zhang X., Yang S.P., Liu W.Q. (2013). Evaluation of the antibacterial activity of patchouli oil. Iran. J. Pharm. Res..

[18] Heo M.H., Kim C., Kim C.H., Ahn C.H., Ahn H.C., Ahn H.Y. (2006). The effects of inhalation of essential oils on the body weight, food efficiency rate and serum leptin of growing SD rats. J. Korean Acad. Nurs..

[19] Kim D.S., Goo Y.M., Cho J.J., Lee J.K., Lee D.Y., Sin S.M., Kil Y.S., Jeong W.M., Ko K.H., Yang K.J. (2018). Effect of volatile organic chemicals in chrysanthemum indicum linné on blood pressure and electroencephalogram. Molecules.

[20] Sundaresan V., Singh S.P., Mishra A.N., Shasany A.K., Darokar M.P., Kalra A., Naqvi A.A. (2009). Composition and comparison of essential oils of pogostemon cablin (blanco) benth. (patchouli) and pogostemon travancoricus bed. Var. travancoricus. J. Essent. Oil Res..

[21] Li Y.C., Xian Y.F., Ip S.P., Su Z.R., Su J.Y., He J.J., Xie Q.F., Lai X.P., Lin Z.X. (2011). Anti-inflammatory activity of patchouli alcohol isolated from Pogostemonis Herba in animal models. Fitoterapia.

[22] Wu J.Z., Liu Y.H., Liang J.L., Huang Q.H., Dou Y.X., Nie J., Zhuo J.Y., Wu X., Chen J.N., Su Z.R. (2017). Protective role of β-patchoulene from Pogostemon cablin against indomethacin-induced gastric ulcer in rats: Involvement of anti-inflammation and angiogenesis. Phytomedicine.

[23] Linck V.D.M., Silva A.L.D., Figueiro M., Piato A.L., Herrmann A.P., Brick F.D., Caramao E.B., Nunes D.S., Moreno P.R.H., Elisabetsky E. (2009). Inhaled linalool-induced sedation in mice. Phytomedicine.

[24] Fonseca D.V.D., Filho C.D.S.M.B., Lima T.C., Almeida R.N.D., Sousa D.P.D. (2019). Anticonvulsant essential oils and their relationship with oxidative stress in epilepsy. Biomolecules.

[25] Batubara I., Suparto I., Sa’diah S., Matsuoka R., Mitsunaga T. (2015). Effects of inhaled citronella oil and related compounds on rat body weight and brown adipose tissue sympathetic nerve. Nutrients.

[26] Suijun W., Zhen Y., Ying G., Yanfang W. (2014). A role for trans-caryophyllene in the moderation of insulin secretion. Biochem. Biophys. Res. Commun..

[27] Fernandes E.S., Passos G.F., Medeiros R., Cunha F.M.D., Ferreira J., Campos M.M., Pianowski L.F., Calixto J.B. (2007). Anti-inflammatory effects of compounds alpha-humulene and(-)-trans-caryophyllene isolated from the essential oil of *Cordia verbenacea*. Eur. J. Pharmacol..

[28] Paula-Freire L.I.G., Andersen M.L., Gama V.S., Molska G.R., Carlini E.L.A. (2014). The oral administration of trans-caryophyllene attenuates acute and chronic pain in mice. Phytomedicine.

[29] Chandra M., Prakash O., Kumar R., Bachheti R., Bhushan B., Kumar M., Pant A. (2017). β-selinene-rich essential oils from the parts of callicarpa macrophylla and their antioxidant and pharmacological activities. Medicines.

[30] Tsuji T., Tanaka S., Bakhshishayan S., Kogo M., Yamamoto T. (2018). Olfactory stimulation modulates the blood glucose level in rats. Int. J. Med. Sci..

[31] Despres J.P. (2006). Is visceral obesity the cause of the metabolic syndrome?. Ann. Med..

[32] Oppenheim R.W. (1996). The concept of uptake and retrograde transport of neurotrophic molecules during development: History and present status. Neurochem. Res..

[33] Yanai R., Yamada N., Inui M., Nishida T. (2006). Correlation of proliferative and anti-apoptotic effects of HGF, insulin, IGF-1, IGF-2, and EGF in SV40-transformed human corneal epithelial cells. Exp. Eye Res..

[34] Arner P. (1995). Differences in lipolysis between human subcutaneous and omental adipose tissues. Ann. Med..

[35] Seo S.M., Jo S.M., Kim M., Lee M., Lee Y., Kang I.H. (2019). Peanut sprout extracts attenuate triglyceride accumulation by promoting mitochondrial fatty acid oxidation in adipocytes. Int. J. Mol. Sci..

[36] Lee H.U., Shin G., Park S.H., Cho H.K. (1999). Insulin resistance and visceral fat obesity in hyperlipidemia. Korean Circ. J..

[37] You M., Fan R., Kim J., Shin S.H., Chung S. (2020). Alpha-linolenic acid-enriched butter promotes fatty acid remodeling and thermogenic activation in the brown adipose tissue. Nutrients.

[38] Zhao H.L., Cho K.H., Ha Y.W., Jeong T.S., Lee W.S., Kim Y.S. (2006). Cholesterol-lowering effect of platycodin D in hypercholesterolemic ICR mice. Eur. J. Phamcol..

[39] Lucka A., Wysokinski A. (2019). Association between adiposity and fasting serum levels of appetite-regulating peptides: Leptin, neuropeptide Y, desacyl ghrelin, peptide YY (1–36), and agouti-related pertein in nonobese participants. Chin. J. Physiol..

[40] Nam S.Y., Kratzsch J., Kim K.W., Kim K.R., Lim S.K., Marcus C. (2001). Cerebrospinal fluid and plasma concentrations of leptin, NPY, and α -MSH in obese women and their relationship to negative energy balance. J. Clin. Endocrinol. Metab..

[41] Simonds S.E., Cowley M.A. (2013). Hypertension in obesity: Is leptin the culprit?. Trends Neurosci..

